# On the Clinical Utility of Hybrid Endoscopy in Crohn's Disease

**DOI:** 10.1002/deo2.70339

**Published:** 2026-05-27

**Authors:** Anastasios Koulaouzidis, Wojciech Marlicz, Ervin Toth

**Affiliations:** ^1^ Department of Gastroenterology Pomeranian Medical University (PUM) Szczecin Poland; ^2^ Department of Clinical Research University of Southern Denmark (SDU) Odense Denmark; ^3^ Department of Surgery Surgical Research Unit Odense University Hospital and Svendborg Hospital Svendborg Denmark; ^4^ Endoklinika The Center for Digestive Diseases Szczecin Poland; ^5^ Department of Gastroenterology Skåne University Hospital Lund University Malmö Sweden

To the Editor:

We read with interest the Original Article by Ito et al. [1] describing same‐day small‐bowel capsule endoscopy (SBCE) combined with ileocolonoscopy in Crohn's disease (CD) remission. While pragmatic, where pan‐enteric capsule endoscopy (PCE) [2] is unavailable, the clinical claims exceed the data. The core claim, i.e., improved SBCE through “standard ileocolonoscopy preparation,” is inadequately characterized. The regimen (agents, doses, timing, boosters) is not fully specified, although cleansing and transit differences underpin the model. Statistically, both groups report a median ileal cleansing score of 2 despite a claimed significant difference (*p* < 0.01). If distributional shifts explain this, full distributions or category proportions ‐not medians alone‐ are required. Without them, the reported improvement risks appear methodological rather than clinically meaningful.

Even accepting modest cleansing benefit, the literature does not support a “more purgative = more useful” narrative [3]. In suspected CD, full PEG ingestion is often poor; although volume correlates with image quality, diagnostic yield may not improve, undermining the implication that better cleansing ensures superior utility [4]. The most consistent signal in SBCE preparation studies is timing ‐shorter intervals improve visibility‐ than regimen intensity [5]. Meta‐analyses further show that “optimal” preparation remains unsettled [3]. If preparation underpins the hybrid proposition, it should be specified and justified as an intervention, not treated as background. Moreover, the “clinical utility” claim is tenuous. In a single‐center retrospective cohort, treatment decisions were left to the physician's discretion, and relapse was defined pragmatically. The resulting Kaplan‐Meier curves are hypothesis‐generating, not proof of improved outcomes, and very small subgroups (only seven de‐escalations) preclude claims of safe de‐escalation.

Finally, the conceptual framing overstates hybridization's uniqueness. If the advantage is pan‐intestinal assessment with biopsies, colonoscopy must demonstrate incremental value beyond capsule findings, particularly as PCE shows strong agreement for SES‐CD severity [2]. Hybrid scheduling may be operationally sensible, but distinct, reproducible clinical benefit remains unproven and requires clearer definition.

## Author Contributions

Anastasios Koulaouzidis conceived and drafted the manuscript. Wojciech Marlicz and Ervin Toth critically revised the manuscript. All authors approved the final version

## Funding

The authors have nothing to report.

## Conflicts of Interest

**Anastasios Koulaouzidis**, **Wojciech Marlicz,** and **Ervin Toth** report industry and editorial relationships as detailed in the submitted ICMJE disclosure forms.
